# Supplementary material for: Genetic connectivity between Atlantic bluefin tuna larvae spawned in the Gulf of Mexico and in the Mediterranean Sea
Source: PeerJ. 2021 Jun 14;9:e11568. doi: 10.7717/peerj.11568 (PMC8210807; doi:10.7717/peerj.11568)
Supplement: Supplemental Information 1 — The minimum number of loci necessary to discriminate between individuals in a population was calculated in R with the “poppr” package (Kamvar, Tabima & Gr̈unwald, 2014). Multilocus genotypes (MLG) at eight microsatellite loci (112 ABFT larvae) were re-sampled 10,000 times to count the number of observed genotypes. [file peerj-09-11568-s001.pdf]

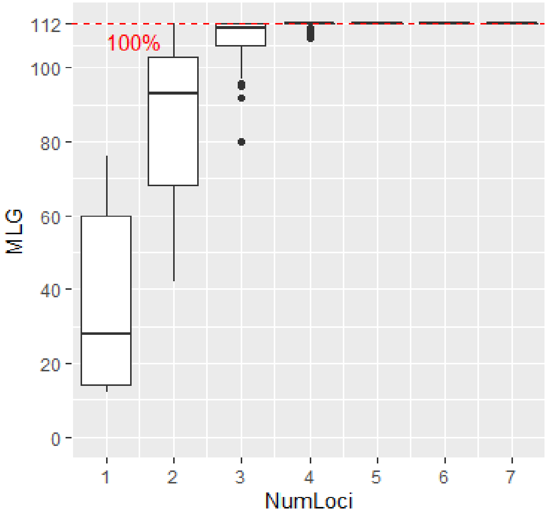

Figure S1. ***Genotype accumulation curve.*** The minimum number of loci necessary to discriminate between individuals in a population was calculated in *R* with the "poppr" package (Kamvar, Tabima & Grunwald, 2014). Multilocus genotypes (MLG) at eight microsatellite loci (112 ABFT larvae) were re-sampled 10.000 times to count the number of observed genotypes.
