# Supplementary material for: Genetic connectivity between Atlantic bluefin tuna larvae spawned in the Gulf of Mexico and in the Mediterranean Sea
Source: PeerJ. 2021 Jun 14;9:e11568. doi: 10.7717/peerj.11568 (PMC8210807; doi:10.7717/peerj.11568)
Supplement: Supplemental Information 2 — Plots obtained with Structure Harvester (Earl & vonHoldt, 2012) from all simulations performed with STRUCTURE v2.3.4 (Pritchard, Stephens & Donnelly, 2000) shown in Fig. 2A. The upper panel shows a plot of the mean likelihood L(K) and variance of estimated probabilities of belonging to a certain cluster per K. The middle pannel plots the mean difference between successive likelihood values of K (L′(K) used to calculated the statistic of Evanno ∆K (deltaK) that is plotted in the lower panel, and can only be obtained for K = 2. [file peerj-09-11568-s002.pdf]

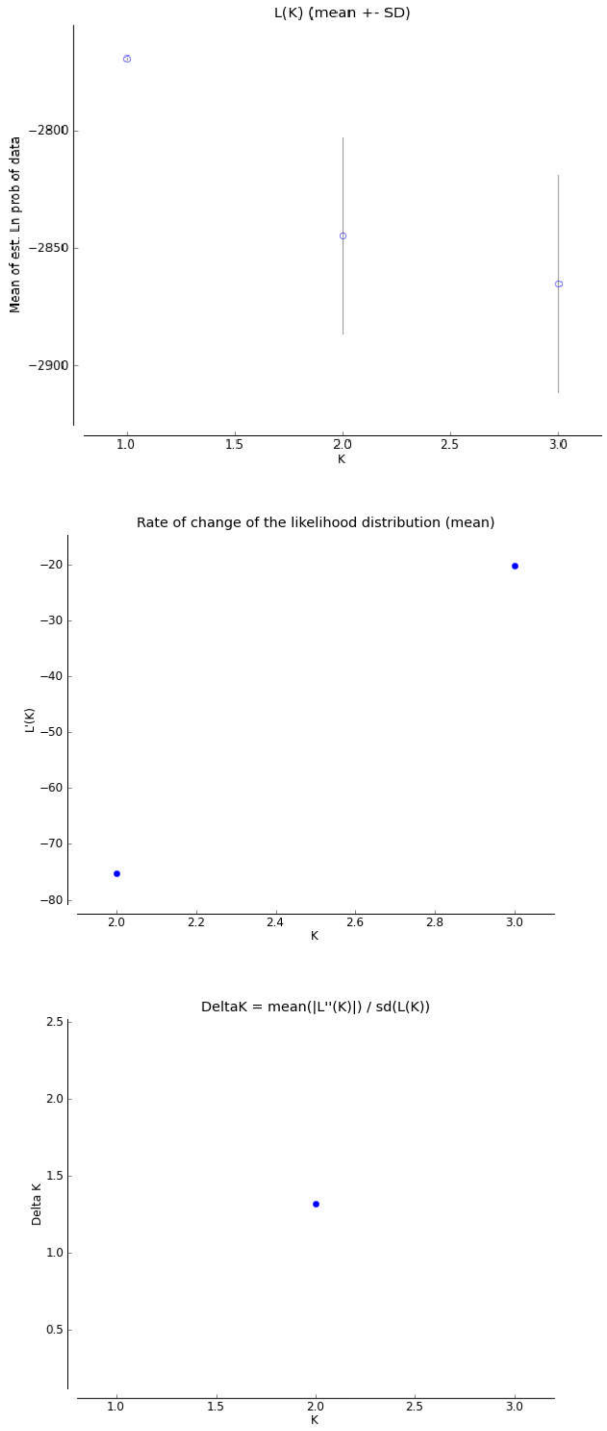

Figure S2. **Exploration of clustering through *Bayesian analysis.*** Plots obtained with *Structure Harvester* (Earl & vonHoldt, 2012) from all simulations performed with *STRUCTURE v2.3.4* (Pritchard, Stephens & Donnelly, 2000) shown in Fig. 2A. The upper panel shows a plot of the mean likelihood L(K) and variance of estimated probabilities of belonging to a certain cluster per *K*. The right plot shows the mean difference between successive likelihood values of K (L´(K) used to calculated the statistic of Evanno *K* (deltaK) that is plotted in the lower panel, and can only be obtained for *K*=2.
