# Supplementary material for: Genetic connectivity between Atlantic bluefin tuna larvae spawned in the Gulf of Mexico and in the Mediterranean Sea
Source: PeerJ. 2021 Jun 14;9:e11568. doi: 10.7717/peerj.11568 (PMC8210807; doi:10.7717/peerj.11568)
Supplement: Supplemental Information 3 — Discriminant Analysis of Principal Components (DAPC) shows differences between clusters through one discriminant function representing diversity in ABFT larvae collected during 2014 in the GOM (grey) and MED (black) spawning areas. Fifty three single nucleotide polymorphisms were genetically characterized in a fragment of the mitochondrial DNA (mtDNA) control region (25 principal components accumulating 0.973 variance in one discriminant function with an eigenvalue of 77.22). [file peerj-09-11568-s003.pdf]

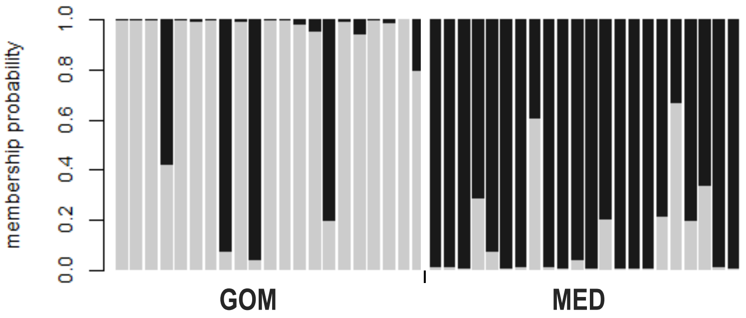

Figure S3. ***Clustering of ABFT larvae genetic diversity at mtDNA control region.*** Discriminant Analysis of Principal Components (DAPC) shows differences between clusters through one discriminant function representing diversity in ABFT larvae collected during 2014 in the GOM (grey) and MED (black) spawning areas. Fifty three single nucleotide polymorphisms were genetically characterize in a fragment of the mitochondrial DNA (mtDNA) control region (25 principal components accumulating 0.973 variance in one discriminant function with an eigenvalue of 77.22).
