# Supplementary material for: Genetic connectivity between Atlantic bluefin tuna larvae spawned in the Gulf of Mexico and in the Mediterranean Sea
Source: PeerJ. 2021 Jun 14;9:e11568. doi: 10.7717/peerj.11568 (PMC8210807; doi:10.7717/peerj.11568)
Supplement: Supplemental Information 4 — DNA was extracted from ABFT fish larvae using the NucleoSpin® Tissue XS (Macherey–Nagel). Multiplex PCR reactions were performed with a Q5® High-Fidelity PCR kit (New England BioLabs, Inc., Ipswich, MA, USA) including 50 ng template DNA and 25 pmol primer mix. The forward primer for each locus was fluorescently labeled for laser detection as indicated. Multiplex PCR conditions were: 97 °C 3 min; 10 cycles (denaturation at 94 °C for 30 s, annealing with 1 °C decrease per cycle from 64 to 54 °C for 30 s for mPCR1 or from 66 to 56 °C for 30 s for mPCR2, extension at 72 °C for 30 s), 25 cycles (94 °C 30 s, 30 s at 54 °C for mPCR1 or at 56 °C for mPCR2, 72 °C 30 s), and final extension at 72 °C for 3 min. Alleles were separated by capillary electrophoresis on an Applied Biosystems 3730xl Genetic Analyzer (Stabvida, Caparica, Portugal), and scored after double manual inspection using GeneMapper® software v4.0 (Applied Biosystems). [file peerj-09-11568-s004.docx]

| **PCR** | **Loci** | **Motif** | **Primers** | **Expected Size (bp)** | **Reference** |
| --- | --- | --- | --- | --- | --- |
| mPCR1 | Ttho 4 | (CA)_n_ | 6-FAM™CCTTCATCTTCAGTCCCATC | 134-164 | Takagi *et al*., 1999. |
|  |  |  | CTGTTCATCTGTTCGCCC |  |  |
|  | Ttho 1 | (GT)_n_ | NED™AAACGCTCCAGGCAAATGAC | 175-189 | Takagi *et al*., 1999. |
|  |  |  | CATAGCACACCCATAGACAC |  |  |
|  | Tth 34 | (CTGT)_7_ | VIC®GATGCCATTTCTCTGTCTATCTG | 99-183 | McDowell *et al*., 2002. |
|  |  |  | AAGCCGTTCCCTCAGTGTC |  |  |
|  | Tth 1-31 | (AC)_11_ | NED™ATGCACAAGTCATTTATCACCT | 96-136 | Clark *et al*., 2004. |
|  |  |  | AGATGCATGGATTACATTCTACC |  |  |
|  | Tth 157 | (CA)_13_ | PET™CAAGAGGCTTAAAGCAAACTC | 123-133 | Clark *et al*., 2004. |
|  |  |  | CATGAATGGGTTCCTTCATC |  |  |
| mPCR2 | Ttho 7 | (CA)_n_ | VIC®ACTGGATGAAAGGCGATTAC | 198-230 | Takagi *et al*., 1999. |
|  |  |  | ACAGAGGAGCATAACAGAAAC |  |  |
|  | Tth 16-2 | (GT)_10_(TACA)_2_ | 6-FAM™TGAGTTCCCAATTACACT | 89-119 | Clark *et al*., 2004. |
|  |  |  | CTGTAGCATCGTCACAGT |  |  |
|  | Tth 208 | (GA)_18_ | PET™GAGAGGGAAAGCAAAGAAG | 148-204 | Clark *et al*., 2004. |
|  |  |  | GTTGAGCTGCTGACACAGA |  |  |
