# Supplementary material for: Genetic connectivity between Atlantic bluefin tuna larvae spawned in the Gulf of Mexico and in the Mediterranean Sea
Source: PeerJ. 2021 Jun 14;9:e11568. doi: 10.7717/peerj.11568 (PMC8210807; doi:10.7717/peerj.11568)
Supplement: Supplemental Information 6 — For each area in which ABFT larvae were collected (Pop), pooled by collection area and all together, the number of samples (N) genotyped, number of alleles found for each locus (Na), Polymorphic Information Content (PIC) and null allele frequency (Null) are indicated. [file peerj-09-11568-s006.docx]

| **Pop^1^** | **GOM** | | | | | | | | | | | | **MED** | | | |
| --- | --- | --- | --- | --- | --- | --- | --- | --- | --- | --- | --- | --- | --- | --- | --- | --- |
|  | **wGOM** | | | | **eGOM** | | | | **GOM (wGOM and eGOM)** | | | |  |  |  |  |
| **Locus** | **N** | **Na** | **PIC** | **Null** | **N** | **Na** | **PIC** | **Null** | **N** | **Na** | **PIC** | **Null** | **N** | **Na** | **PIC** | **Null** |
| **Tth208** | 30 | 16 | 0.9 | 0.0 | 30 | 21 | 0.9 | 0.0 | 60 | 23 | 0.9 | 0.0 | 50 | 19 | 0.9 | 0.0 |
| **Tth1-31** | 32 | 14 | 0.9 | 0.0 | 30 | 13 | 0.9 | 0.0 | 62 | 15 | 0.9 | 0.0 | 50 | 15 | 0.9 | 0.0 |
| **Ttho7** | 27 | 12 | 0.8 | 0.0 | 28 | 14 | 0.8 | 0.0 | 55 | 14 | 0.8 | 0.0 | 50 | 12 | 0.8 | 0.0 |
| **Tth34** | 32 | 12 | 0.7 | 0.0 | 30 | 13 | 0.6 | 0.0 | 62 | 15 | 0.7 | 0.0 | 50 | 16 | 0.8 | 0.0 |
| **Ttho4** | 32 | 9 | 0.7 | 0.0 | 30 | 9 | 0.7 | -0.1 | 62 | 10 | 0.7 | 0.0 | 50 | 10 | 0.7 | 0.1 |
| **Ttho1** | 32 | 7 | 0.6 | 0.1 | 30 | 4 | 0.5 | 0.1 | 62 | 8 | 0.6 | 0.1 | 50 | 6 | 0.6 | 0.2 |
| **Tth157** | 30 | 6 | 0.6 | 0.0 | 30 | 5 | 0.4 | 0.1 | 60 | 6 | 0.5 | 0.0 | 50 | 6 | 0.5 | -0.1 |
| **Tth16-2** | 24 | 8 | 0.5 | 0.3 | 23 | 4 | 0.5 | 0.4 | 47 | 9 | 0.5 | 0.3 | 50 | 3 | 0.4 | 0.3 |
| **Mean** | 30 | 11 | 0.7 | 0.0 | 29 | 10 | 0.7 | 0.1 | 59 | 13 | 0.7 | 0.1 | 50 | 11 | 0.7 | 0.1 |
| **SE** | 1 | 1 | 0.0 | 0.0 | 1 | 2 | 0.1 | 0.1 | 2 | 2 | 0.1 | 0.0 | 0 | 2 | 0.1 | 0.0 |

^1^Pop refers to each area in which ABFT larvae were collected
