# Supplementary material for: Genetic connectivity between Atlantic bluefin tuna larvae spawned in the Gulf of Mexico and in the Mediterranean Sea
Source: PeerJ. 2021 Jun 14;9:e11568. doi: 10.7717/peerj.11568 (PMC8210807; doi:10.7717/peerj.11568)
Supplement: Supplemental Information 7 — For each spawning area replicate datasets excluding missing data were analyzed with COLONY software. Average number and standard deviation are shown for each parameter. N indicates the number of multilocus genotypes (individuals) included in the sibship analysis. Linking unrelated offspring through pedigree results in a number of clusters with average maximum (Nmax) and minimum (Nmin) individuals reported per cluster. Inferred mothers, fathers and families are indicated together with number of full siblings (Full sib) and average associated probability (Full sib p). [file peerj-09-11568-s007.docx]

| **Sibship^1^** | **N** | **Clusters** | **N_max_** | **N_min_** | **Mothers** | **Fathers** | **Families** | **Full sib** | **Full sib p** |
| --- | --- | --- | --- | --- | --- | --- | --- | --- | --- |
| **GOM** | 48 ± 5 | 7 ± 1 | 22 ± 6 | 1 ± 1 | 22 ± 1 | 24 ± 2 | 45 ± 4 | 10 ± 5 | 0.174 ± 0.105 |
| **MED** | 50 ± 0 | 7 ± 2 | 17 ± 3 | 1 ± 1 | 22 ± 3 | 24 ± 1 | 47 ± 1 | 8 ± 4 | 0.228 ± 0.135 |

**^1^**Replicate data consisted of triplicate multilocus genotypes at 8 microsatellite loci, and for 7 and 6 loci (excluding Tth16-2 and also Ttho1 due to null allele presence).
